# Development of Al-Mg_2_Si Alloy Hybrid Surface Composites by Friction Stir Processing: Mechanical, Wear, and Microstructure Evaluation

**DOI:** 10.3390/ma16114131

**Published:** 2023-06-01

**Authors:** R. Raja, Ragavanantham Shanmugam, Sabitha Jannet, G. B. Veeresh Kumar, N. Venkateshwaran, K. Naresh, Monsuru Ramoni

**Affiliations:** 1 Department of Mechanical Engineering, Karunya Institute of Technology and Sciences, Coimbatore 641114, India; 2 School of Engineering, Math and Technology, Navajo Technical University, Crown Point, NM 87313, USA; 3 Department of Mechanical Engineering, National Institute of Technology-Andhra Pradesh, Tadepalligudem 534101, India; 4 Department of Mechanical Engineering, Rajalakshmi Engineering College, Chennai 600125, India; 5 Department of Chemical Engineering and Materials Science, University of Southern California, Los Angeles, CA 90089, USA

**Keywords:** FSP, UTS, elongation, microhardness, wear rate, SEM, TEM

## Abstract

Surface composites are viable choices for various applications in the aerospace and automotive industries. Friction Stir Processing (FSP) is a promising method for fabricating surface composites. Aluminum Hybrid Surface Composites (AHSC) are fabricated using the FSP to strengthen a hybrid mixture prepared with equal parts of Boron carbide (B_4_C), Silicon Carbide (SiC), and Calcium Carbonate (CaCO_3_) particles. Different hybrid reinforcement weight percentages (reinforcement content of 5% (T1), 10% (T2), and 15% (T3)) were used in fabricating AHSC samples. Furthermore, different mechanical tests were performed on hybrid surface composite samples with different weight percentages of the reinforcements. Dry sliding wear assessments were performed in standard pin-on-disc apparatus as per ASTM G99 guidelines to estimate wear rates. The presence of reinforcement contents and dislocation behavior was investigated using Scanning Electron Microscopy (SEM) and Transmission Electron Microscopy (TEM) studies. The results indicated that the Ultimate Tensile Strength (UTS) of sample T3 exhibited 62.63% and 15.17% higher than that of samples T1 and T2, respectively, while the Elongation (%) of T3 exhibited 38.46% and 15.38% lower than that of samples T1 and T2, respectively. Moreover, it was found that the hardness of sample T3 increased in the stir zone compared to samples T1 and T2, owing to its higher brittle response. The higher brittle response of sample T3 compared to samples T1 and T2 was confirmed by the higher value of Young’s modulus and the lower value of Elongation (%).

## 1. Introduction

Aluminum (Al) surface composites are progressively replacing conventional alloys for applications in the automotive, aircraft, and shipbuilding industries [1]. The FSP is a very effective method to improve the surface properties of metals among numerous production methods. It is a solid-state procedure used to modify surface properties [2,3]. The traditional issues connected with a liquid process, such as the wettability of the reinforcing particles with the molten metals, are not present in this process [4]. The addition of an equal weight percentage (wt%) of B_4_C and Titanium Diboride (TiB_2_) will enhance microhardness and tensile properties. The development of a tribolayer during sliding improved the wear resistance of the hybrid surface composite [5]. Uniform finer grains were obtained by reinforcing B_4_C and TiB_2_ nanoparticles in the Al7075 alloy [6]. The wear resistance was enhanced by about 61% due to the addition of reinforcement [7]. The microhardness and wear properties of wrought Al7075 alloy with SiC and Boron Nitride (BN) nanoparticles were investigated. The wear resistance increased by 61 percent, and microhardness increased by 45 percent in the manufactured composites [8]. Using mono and mixed additions of ceramic and metal particles as reinforcement, the corrosion and microstructural evolutions of surface composites were examined after the fabrication of surface composites by FSP. Mono-reinforced surface composite displayed decreased corrosion, whereas the mixed reinforcement surface composite showed a galvanic effect [9].

The Al6061 alloy was reinforced with Zirconium dioxide (ZrO_2_) from 0 to 10% and ZrO_2_ and Graphene together as two different Metal Matrix Composites (MMCs). The comparative study showed that ZrO_2_-reinforced composites exhibited enhanced mechanical properties, whereas composites reinforced with ZrO_2_ and Graphene showed improved wear properties [10]. The surface of the composites was synthesized using Iron Oxide (Fe_3_O_4_), Al powder as reinforcement, and Al1050 alloy as the substrate. The mean grain size of the matrix decreased. An increase aided the metal matrix’s dynamic restoration in high-angle grain boundaries [11]. To improve the microstructure and mechanical performance of AA6061-15 wt% Al_3_Ni MMCs, FSP was used as a secondary procedure. The following method resulted in a significant reduction in grain size and the removal of casting faults, such as porosity [12]. Molybdenum as the metallic reinforcement and B_4_C as the ceramic reinforcement with Al1050 as the substrate was combined to fabricate metal matrix surface composites; the mono reinforcement showed a reduction in corrosion rate, whereas the combined reinforcement showed a galvanic effect [8].

The FSP was implemented to reinforce the Al5083 surface with SiC and Alumina (Al_2_O_3_) microparticles to obtain surface composites. The obtained results noticed an enhancement in the microhardness, tensile strength, and wear properties [13]. The FSP is used to reinforce Silicon Nitride (Si_3_N_4_) and Aluminum Nitride (AlN) with the base metal Al2024. By eliminating flaws caused by metal melting, the hardness, corrosion resistance, microstructure formation, wear resistance, and durability of Al2024 were improved [14]. FSP used Al_2_O_3_ micro and nanoparticles to strengthen the Al356 alloy surface. The surface composites’ microhardness and elastic modulus characteristics were enhanced [15]. Another experiment employed several process settings to reinforce Al1050 with SiC particles. As the traverse and rotation speeds were increased, the SiC particles were better distributed across the substrate. The base Al alloy microhardness was increased three times [16]. The atomized Al powder and SiC powder were cold compacted to produce billets, and FSP then processed these billets. The process refined the grain structure leading to enhanced tensile behavior [17].

Al5083 alloy and Al6061-T651 were reinforced with SiC and Al_2_O_3_ hybrid particles, and improved tensile and wear properties were achieved [18]. By reinforcing an Al6061-T651 alloy plate with Al_2_O_3_ sub-micron-size and SiC particles, a surface composite up to 3 mm thick was formed, and the hard phase was detected at a 20–30% concentration. Surface composites with steel as the bearing metal reduced friction and wear by 40% and 90%, respectively, compared to the base metal Al surface [19]. The Taguchi method of DOE was used to optimize parameters, such as rotational tool speed and reinforcement percentage to achieve enhanced wear and tensile characteristics. According to microstructure photographs, the SiC and Al_2_O_3_ reinforcements were equally spread in the nugget zone [20]. The hybrid surface composite was created by reinforcing the SiC and MoS_2_ particles on the matrix metal Al356 alloy surface. The process settings were 1600 rpm rotational speed, 50 mm/min travel speed, and a 3° tool tilt angle to obtain the same results. The nugget zone featured a uniform distribution of reinforcement particles and a MoS_2_-rich Mechanically Mixed Layer (MML) on top of the worn surface, which improved wear properties [21]. According to microstructures, the reinforcement particles (Gr, Al_2_O_3_, and SiC) are evenly distributed in the nugget zone of all surface hybrid composites. As indicated by micrographs, the reinforcement particles (SiC, Gr, and Al_2_O_3_) were equally distributed in the nugget zone of all surface hybrid composites, and the pinning effect of hard SiC and Al_2_O_3_ particles strengthened the microhardness of Al–Al_2_O_3_/SiC surface hybrid composites [22,23].

The degree of spreading, mixing, and dispersion was identified as three distinct stages of particle distribution [24]. The Fretting fatigue to abrasion was predominantly observed in the wear mechanism derived from wear debris analysis and worn-out track micrographs. The impact of tool traverse speed and volume percent of reinforcement on rotational tool speed, Titanium Carbide (TiC), Gr, and the interaction effect between rotational tool speed and TiC/Gr hybrid ratio [25]. On the matrix surface, the influence of process factors, such as rotational tool speed, traverse speed, and reinforcement ratio on the B_4_C and Al_2_O_3_ was investigated [26,27].

This research aimed to examine and analyze the dynamic material flow and numerous property and microstructure evaluations of Al hybrid surface composite. The mechanisms involved in property evaluation are analyzed in detail using SEM, including Energy dispersive X-ray spectroscopy (EDS), optical microscopy (OM), and TEM techniques. In this regard, there is a paucity of literature on reinforcements with three different elements. The synergistic effect of three different reinforcement particles as a hybrid is evaluated. The effect of the combined effect of the particles is studied. As a result, this serves as a foreground for the current study and contributes to future research along with the same principles.

## 2. Materials and Methods

### 2.1. Materials and Fabrication of Composite Samples

**Figure 1:**
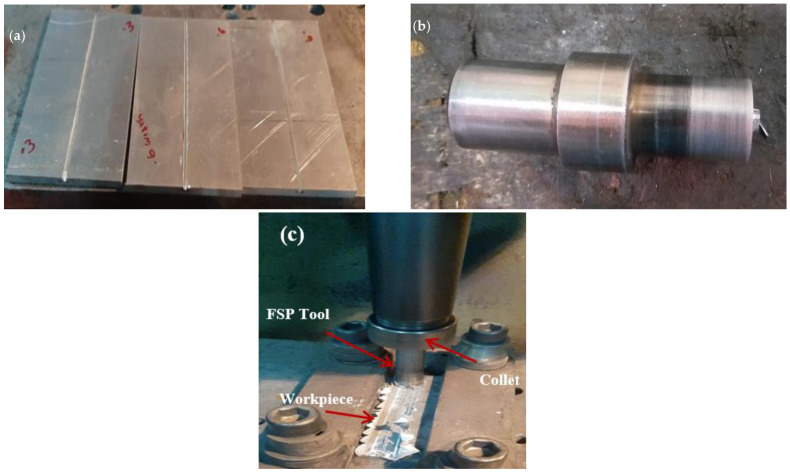
Steps involved in the fabrication process: Preparation of Aluminum plate with grooves (**a**), assembling the FSP tool (**b**) in the Friction Stir Processing setup (**c**).

The matrix metal used was a rolled Al-Mg_2_Si Alloy plate with a dimension of 120 mm × 75 mm × 6 mm acquired from Metal Mart in Coimbatore, India. The elemental composition of the matrix metal is given in Table 1. The B_4_C powder with an average particle size of 50 microns, SiC powder with the average particle size of 50 microns, and Calcium Carbonate (CaCO_3_) powder with the average particle size of 100 microns. An equal proportion of the reinforcement particles were mixed in acetone solution using a magnetic stirrer for 6 h. The particles were then dried for around 24 h. These dry particles were then crushed within Al plate grooves that had been cut. The groves were machined to a depth of 3.5 mm and widths of 0.6 mm, 0.9 mm, and 1.2 mm, as shown in Figure 1a. This corresponds to three different combinations of samples which were fabricated based on the addition of different weight percentages (5, 10, and 15 wt%) of reinforcement contents in the metal matrix, i.e., the sample T1 has 5 wt% reinforcement content, the sample T2 has 10 wt% reinforcement content and the sample T3 has 15% of reinforcement content, as given in Table 2. The tool was made of hardened steel H13 with an 18 mm shoulder diameter, 1.7 mm pin height, and a 6 mm diameter, as shown in Figure 1b. For FSP, pin, and pinless devices were utilized. The top surface layer of the groove was sealed with a pinless tool, and the matrix and reinforcement were equally mixed with a pin tool. To prevent movement during the FSP, the workpiece is fastened to the fixture, as shown in Figure 1c. The tool rotated clockwise in the groove containing B4C, SiC, and CaCO_3_ Powder. The exact process was completed thrice. First, the tool was rotated without the pin, which is considered a capping process. For all passes, the common process parameters (a traverse speed of 35 mm/min and a rotating speed of 1200 rpm) were used.

The second pass is carried out by using a tool with a pin. Hence, the particle reinforcement occurs on the Al plate’s surface from the second pass onward. The third pass is where the tool with the pin is directed in the counter direction, and it is stopped in the same spot where it started. The samples were machined using the Electrical Discharge Machining (EDM) process for mechanical and microstructural studies.

### 2.2. Experimental Details

#### 2.2.1. Mechanical Characterization

A standard tensile sample was cut as per the ASTM E8 standard, i.e., a dog bone shape sample with a gauge length of 20 mm. The tensile test samples were cut from the stir zone parallel to the tool traverse direction. The tensile test was carried out at a crosshead speed of 1.3 mm/min using the universal testing machine (TMC-Chennai, CUTM-50 kN, Chennai, India).

The microhardness test was carried out using Mitutoyo, Kanagawa, Japan HM113 machine, and the load applied was 50 g for 10 s. The sample dimensions, such as length and width used for the Vickers hardness test, were 30 mm and 6 mm, respectively. A diamond indenter with an angle of 136° between opposite faces was used.

A dry sliding wear test was carried out as per ASTM G99 standard [28] using DuCom Instrument (Make TR20-LE), Bangalore, India. The sliding distance used was 2500 m, the velocity applied was 1.5 m/s, and the load applied was 25 kg.

#### 2.2.2. Fractography Analysis

Gatan sample preparation equipment was used for the sample preparation of fractography analysis. The Minitom low-speed diamond saw was employed to slice the sample up to 500 microns. A disc punch was used to further obtain 3 mm diameter discs from the samples. Furthermore, a disc punch was used to bring the thickness of the 3 mm discs up to around 80 microns. Precision Ion Polishing System (PIPS) with Liquid Nitrogen cold stage and the auto terminator was used to reduce the sample thickness to 10 microns.

Before being analyzed with a transmission electron microscope (TEM), the samples were reduced to a thickness of 100 microns, then punctured into spheres in the stir zone. The TEM analysis was performed using a JEOL JEM2100 High-Resolution TEM with a 200 kV working voltage (Tokyo, Japan). The scanning electron microscopy and the EDS analysis of the reinforcement particles were studied using the FESEM instrument (Carl Zeiss, White Plains, NY, USA). This instrument can investigate structures as small as 1.5 nm and magnify objects up to 5 lakh times that size.

The samples for the optical microscopy (OM) were prepared by polishing using Emery paper of different grades (800, 1000, 1200, 1400, and 1600 μm). After that, the samples were further polished using a twin-disc polishing machine. The samples were then etched with Keller’s reagent as the etchant. Furthermore, the microstructure investigation was carried out using an Optical Microscope to make QS Metrology XJL17 with a magnification of up to 400×.

## 3. Results and Discussion

### 3.1. Energy Dispersive X-ray Spectroscopy (EDS) Analysis

**Figure 2:**
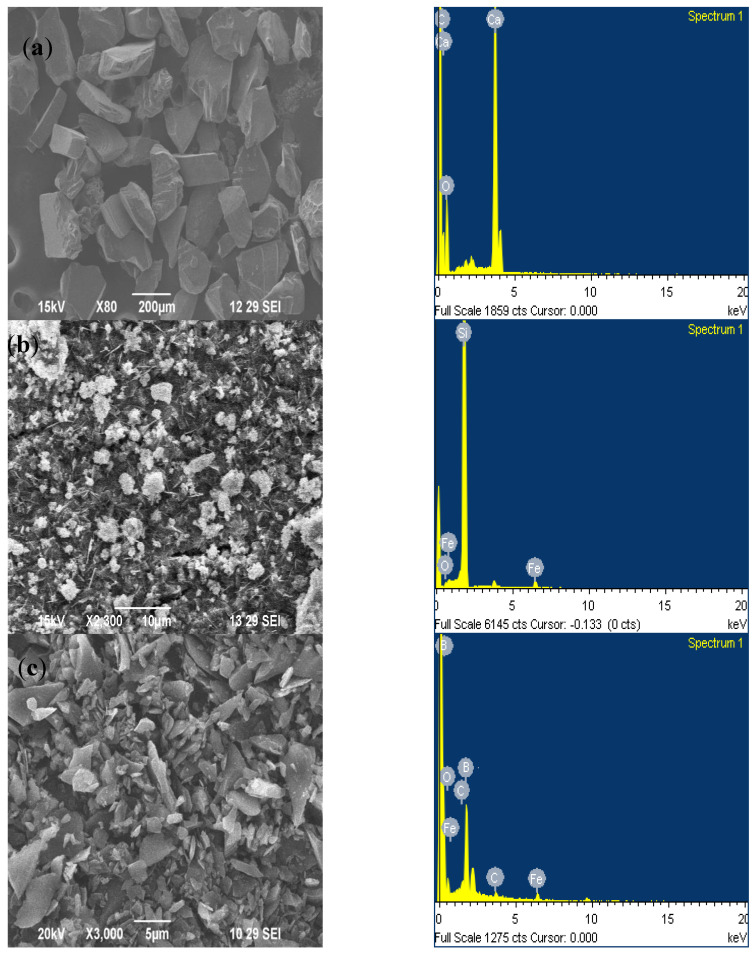
Reinforcement particles with EDS Analysis: (**a**) CaCo_3_, (**b**) SiC, and (**c**) B_4_C.

Particles were analyzed to identify the elemental composition, as shown in Figure 2. The EDS maps in Figure 2a show the existence of calcium in two high peaks and carbon. Figure 2b indicates the presence of silicon at the highest peak. Figure 2c shows the presence of boron carbide at the highest peaks. Thus, the particles were identified, which were used as reinforcements in fabricating the metal matrix composite samples.

### 3.2. Effect of Hybrid Reinforcement on the Tensile Properties

**Figure 3:**
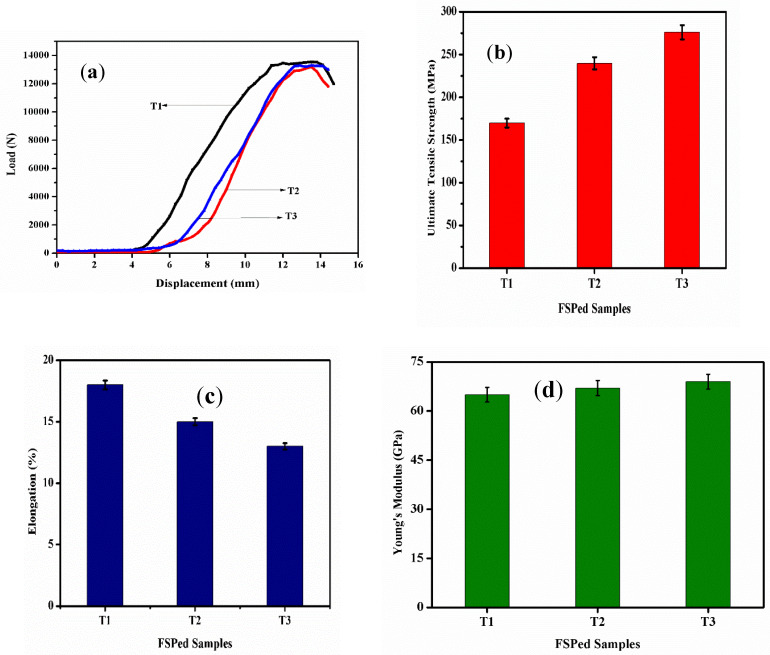
Effect of hybrid particles on the tensile properties of surface composites: (**a**) Load—Displacement, (**b**) UTS, (**c**) Elongation (%), and (**d**) Young’s Modulus.

The tensile properties of the surface hybrid composites with increasing hybrid reinforcement are shown in Figure 3a–c. Grain boundary sliding, and dislocation motion might be limited by reinforcement particles, such as B_4_C, SiC, and CaCO_3_. Furthermore, a poor interfacial bond between the reinforcement particles and the matrix is observed [22]. It is observed from Figure 3b that the ultimate tensile strength of sample T3 is 62.63% and 15.17% higher than that of samples T1 and T2, respectively. From Figure 3c, it is observed that the Elongation (%) of T3 is 38.46% and 15.38% lower than that of samples T1 and T2, respectively. Incompatible deformation between the plastically deformed matrix and the stiff reinforcing particles causes dislocations in the composites. The addition of reinforcing particles inhibits elongation by increasing the effective slip distance [24]. The applied tensile load is distributed throughout the Al matrix via comparably tougher hybrid reinforcement. The Young’s Modulus is the amount of rigidity in the Elastic region, as shown in Figure 3d for different samples. It was obtained from the slope of the linear curve from the stress–strain curve.

Dislocation movement is slowed by creating strain fields caused by changes in the thermal expansion coefficient. The enhanced load transfer due to the significant interfacial bonding could also contribute to the achieved properties. Pore removal makes the composite denser and gives it more room to resist tensile loads. Tensile strength is increased by strain fields created by deformation-induced dislocations [8]. The fact that grain development slows as the fraction of hybrid reinforcement particles increases aligns with the phenomena of increased mechanical strength with increasing reinforcement particle addition. The addition of hybrid particles increased yield strength, which resulted in improvements in the hardness value of the surface composite and a reduction in grain size. The presence of hybrid particles in the metal matrix reduced dislocation movement and reduced the ductility of the matrix [29,30,31].

**Figure 4:**
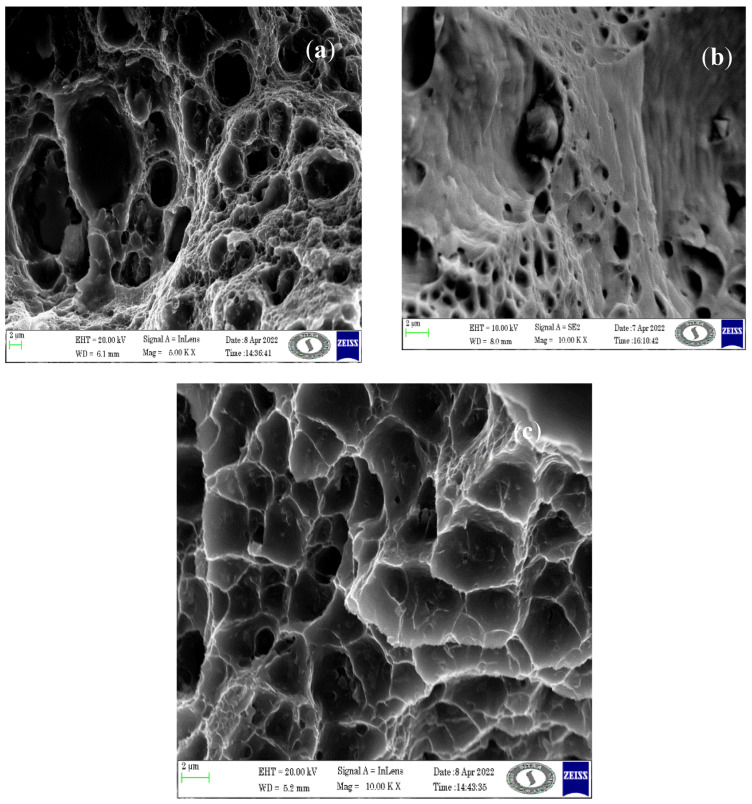
FESEM investigation in fractured samples (**a**) T1, (**b**) T2, and (**c**) T3.

Fracture morphologies of the tensile samples are shown in Figure 4a–c. Sample T1 shows deeper dimples and thicker tear edges, indicating a ductile fracture. In sample T2, hybrid particles are present in the pores indicating a brittle fracture. In sample T3, the quasi-cleavages are noted, which indicates a brittle fracture. Large, deep, and closely spaced dimples indicate high ductility and energy absorption capacity, while small, shallow, and widely spaced dimples indicate lower ductility.

### 3.3. Effect of Hybrid Reinforcements on Microhardness Properties

**Figure 5:**
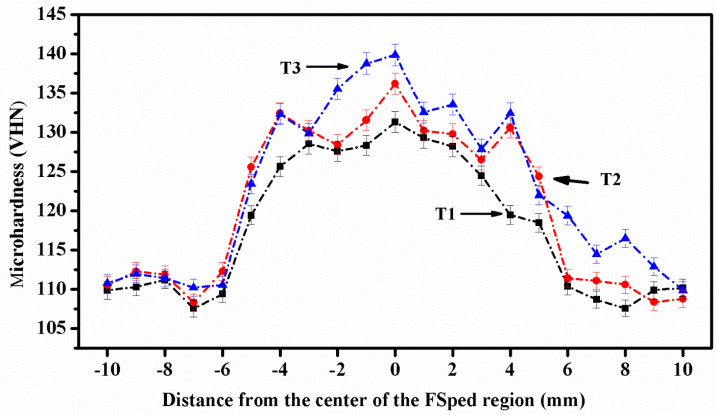
Effect of Percentage of Hybrid reinforcement on Microhardness.

Figure 5 shows the microhardness VHN for the entire processed zone. Stirred samples were split into base alloy and Heat-Affected Zones (HAZ) based on their distance from the stirred zone to evaluate the microhardness profile throughout the stirred area. After that, the measurements from each zone were merged to form a profile. In the HAZ, the treated samples have a low hardness. High-temperature qualities alter the behavior of the softened matrix metal and the coarsening effect caused by particle reinforcement. The DRX effect and the matrix grain size strengthening effect cause grain size reduction in the stir zone, resulting in higher hardness than the HAZ zone.

On the other hand, the hardness effect on the stir zone is a result of a combination of particle distribution and grain size strengthening mechanisms [32], and the dominance of precipitate solution hardening. The hybrid zone has a higher hardness than the base metal, as shown in Figure 5. Microstructural refinement, homogeneity, and densification are responsible for this [21,33]. The FSP considerably increases the Al substrate’s hardness. Increased hardness is anticipated due to the uniform dispersion of SiC_p_ and B_4_C particles with extremely high hardness and considerable microstructural change caused by FSP [34]. The presence of ceramic reinforcements is responsible for the increase in hardness. The uniform dispersion of reinforcing particles in the matrix and the Orowan strengthening mechanism are two critical elements for the increase in hardness [35]. The microhardness fluctuates since the heat varies in different zones of the material. In the stir zone, the change is insignificant as the string of the metal occurs, which lead to an increase in hardness [36]. The strain-hardening behavior of the applied mechanical load challenges the heat generated in the thermos-mechanical affected zone. Consequently, the hardness variation is significant [37].

The excellent distribution deters dislocation movement resulting in the material’s hardness. According to the Hall–Patch connection, metal mechanical characteristics are inversely related to grain size. The grains in the composite have undergone a great deal of refinement. The composite’s hardness is improved by the refined grains. The effect of those factors is amplified as the volume fraction rises. Work hardening occurs because of dislocation interactions and their accumulation during deformation. The accumulated dislocations contribute to increased microhardness due to the strain-hardening effect.

Furthermore, as the volume fraction rises, the average inter-particle distance falls, increasing the contact between the Al alloy matrix and hybrid particles [38]. As the hybrid particle composition increases, the pinning effect becomes more robust. Ceramic particles can operate as load barriers, minimizing plastic deformation because there is less direct contact between the pin and the counterpart disc. The wear rate is decreased as the hybrid reinforcement content is increased. This is because surface composite layers containing more hybrids have a higher hardness [13]. Some strengthening mechanisms that enhance hardness after FSP are dispersion hardening, grain refining, and dislocation interaction with non-shearable carbide particles [39,40].

The composite that included friction stir processing (FSP) and hybrid particles generally increased the surface hardness of the. Hybrid composites.

The zones of the specimens, such as the base alloy zone and the heat-affected zone (HAZ), were separated based on how far away from the stirred zone they were, and the microhardness profile over the stirred zone was also looked at. A profile was made after the microhardness of each zone was assessed (see Figure 5). The profile revealed a rise in hardness as the agitated zone was approached. From the far edge of the HAZ, away from the stirred zone to the other edge of the HAZ close to the stirred zone, hardness results rose consistently.

### 3.4. Effect of Hybrid Reinforcement on Wear Behaviour

**Figure 6:**
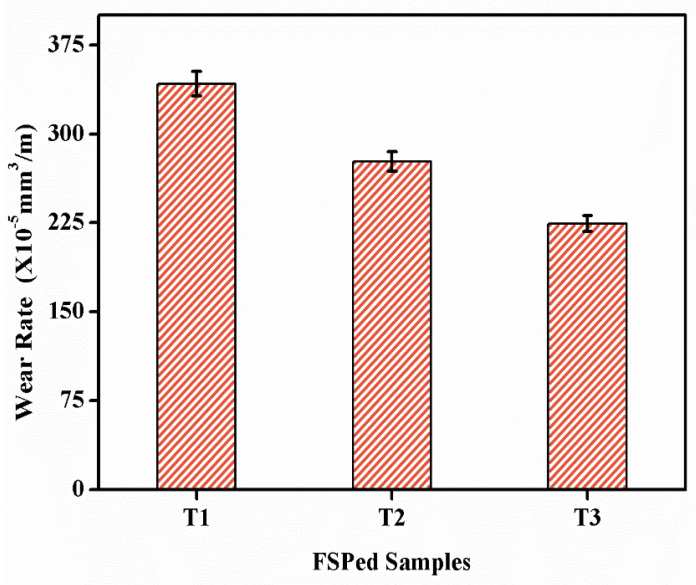
Effect of percentage hybrid reinforcement on the wear rate.

By incorporating hybrid particles, the material’s abrasive wear resistance was improved while the plowing tendency was minimized, as shown in Figure 6. The surface composite layers appear to have lesser abrasiveness than the underlying alloy. This could be due to the material’s increased hardness and the presence of reinforcing ceramic particles, which can help decrease plastic deformation [13]. The wear test results indicate that it has the lowest wear rate because reinforcement particles are used to strengthen the composite. Hardness and wear rate have an inverse connection, according to Archard’s wear law [41]. The presence of reinforcement would have resulted in stress concentration at the reinforcement/matrix interface as dislocations piled up, allowing fracture nucleation and propagation to occur more quickly. By delaying the initiation and propagation of cracks, the homogeneous distribution of refined reinforcement would have improved the wear resistance of Al-Mg_2_Si alloys. The wear rate decreased by 21.14% in the T2 sample compared to sample T1, and a 20.90% decrease was observed in the T3 sample compared to the T2 sample [42,43].

**Figure 7:**
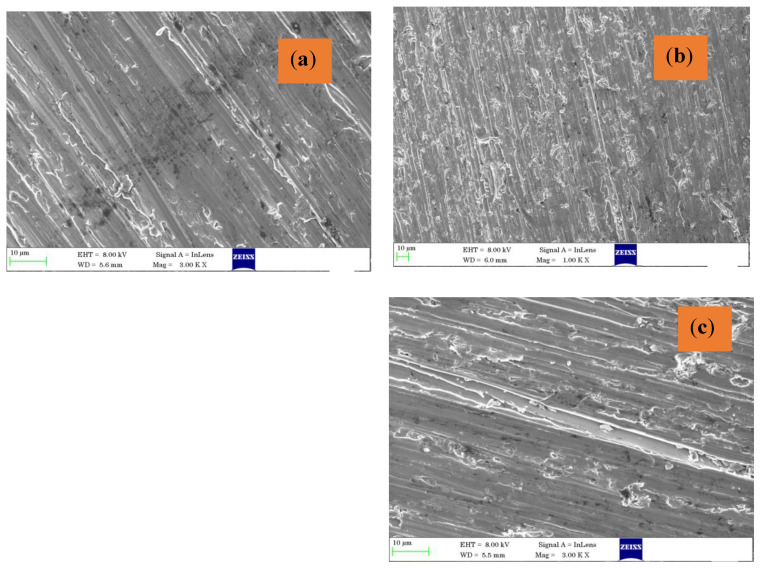
Worn surface in different types of samples: (**a**) T1, (**b**) T2, and (**c**) T3.

The worn surface morphology is shown in Figure 7a–c. The worn surfaces have indicated adhesion wear as the debris has again placed itself on the surface, which leads to wear in sample T3.

The non-reinforcement behavior of the particles and big grains weakened the composite matrix, resulting in the sample with the lowest wear resistance [21]. Another reason for these differences is that hybrid particles can withstand the imposed stress while resisting the Al matrix’s plastic deformation. Moreover, the thermal expansion coefficients of Al and reinforcing particles vary. This deviation adds to the number of dislocations [44,45].

Due to the presence of hybrid particles, the actual contact surface between the rubbing pin and the spinning disc decreases. During sliding, hybrid particles take on the regular burden right away. Due to the load-carrying action, the coefficient of friction at the contact surface is lowered. The proper bonding of hybrid particles to the Al matrix avoids particle pullout from the surface and thus decreases the wear rate [46,47,48].

The wear rates of different specimens varied based on their resistance to wear, which was influenced by the composition of the alloys and the amount of nanoparticles present. By subjecting the AlMg_2_Si alloy to Friction Stir Processing (FSP), its wear rate was reduced. This improvement was primarily due to the FSP’s impact on refining the grain structure. The composite alloys T2 and T3 demonstrated a significant deterioration in wear rate. However, the wear rate of T1 can be attributed to the lack of reinforcement from large grains and particles, which weakened the composite matrix.

The wear rates of hybrid composites containing different volume fractions of particles were analyzed. The hybrid composites showed a decrease in wear rate. Notably, the inclusion of hybrid particles had a significant impact on the reduction in wear rate in the hybrid composite materials. This effect can be observed in Figure 6. Therefore, the wear resistance was positively influenced by both the Friction Stir Processing (FSP) technique and the addition of hybrid particles [49]. It is worth noting that the wear resistance was affected by the behavior of particles and large grains, which may have softened the composite matrix.

### 3.5. Microstructural Analysis

**Figure 8:**
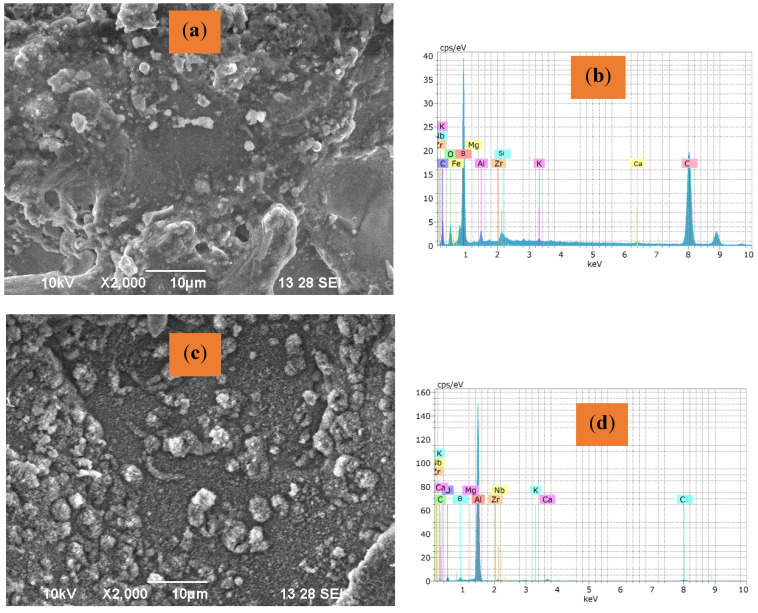

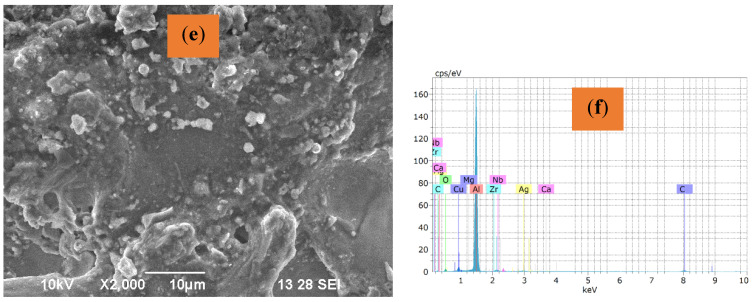
SEM Analysis of Particle distribution: (**a**) Sample T1 (**b**) EDS of Sample T1; (**c**) Sample T2 (**d**) EDS of Sample T2; (**e**) Sample T3 (**f**) EDS of Sample T2.

The reinforcement particles are scattered over the metallic matrix, and their respective elemental distribution is illustrated in the micrographs in Figure 8a–f. Reinforcing particles segregate at grain boundaries in stir casting and other liquid metallurgical processing methods [50]. When the matrix material does not melt, the effects of solidification are eliminated. The idea of reinforcing particles roaming smoothly owing to a density difference inside the plasticized material before forging is eliminated. Consequently, Fly Ash particles were reinforced adequately in the composite [51]. The shattering of hybrid particles, which resulted in a refined microstructure, could have caused severe plastic deformation, a fundamental property of FSP [39].

**Figure 9:**
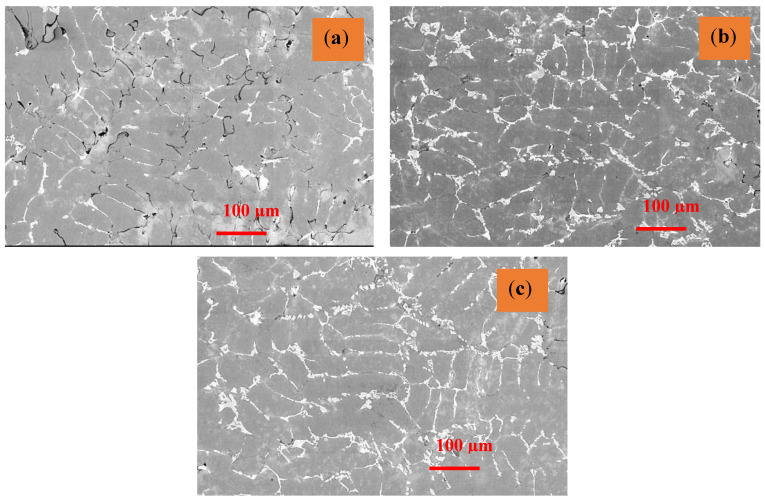
Microstructure investigation in the stir zone in different types of samples through SEM: (**a**) T1—5 wt%, (**b**) T2—10 wt%, (**c**) T3—15 wt%.

**Figure 10:**
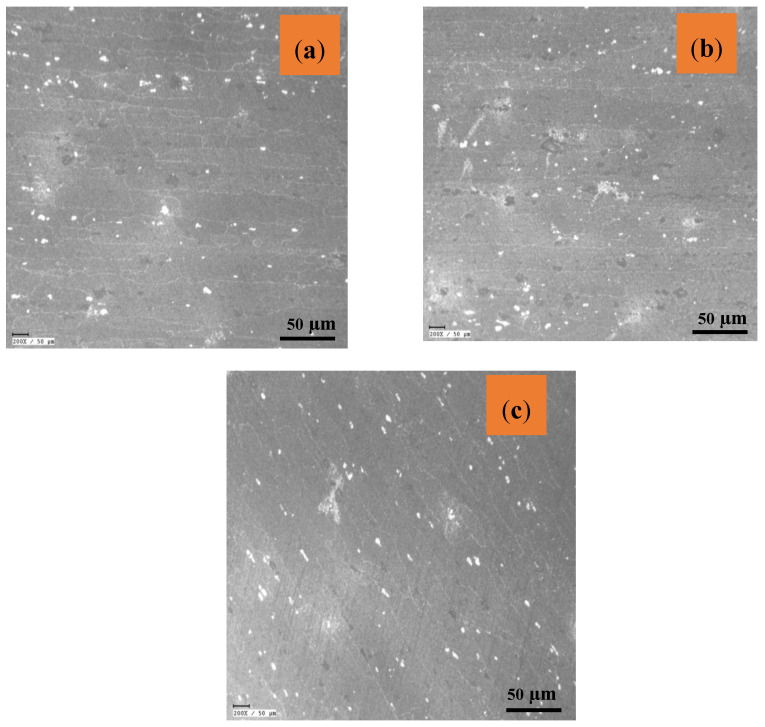
Optical Micrographs of particle distribution in the stir zone, (**a**) T1—5 wt%, (**b**) T2—10 wt%, (**c**) T3—15 wt%.

The strain created during FSP has been much larger than the strain developed during other severe plastic deformation procedures [52]. The inclusion of reinforcing particles, which causes a higher rate of dislocation formation, has been linked to this behavior. Differential thermal contraction between the reinforcing particles and the matrix and the Orowan process have been identified as the primary causes of increased dislocation density in the composite layer [53]. The sub-grains capable of converting to a grain must have a large misorientation angle relative to the nearby deformed material for Continuous Dynamic Crystallization [54,55,56]. After FSP, grain orientation changes significantly, as shown in SEM images in Figure 9a–c. The FSP-processed composite has a lot of low-angle grain boundaries. The sub-grain boundaries formed by dislocation rearrangement are displayed in Figure 10a–c. During the deformation process, dislocations are gradually integrated into sub-grain barriers, increasing their orientation, and transforming them into low-angle grain boundaries [57]. During the FSP process, Al is predominantly subjected to significant plastic deformation. The grains have been observed to be oriented in a similar direction in Sample T1 in Figure 9a, whereas the grain boundaries in sample T2 (Figure 9b) have been oriented in different directions. However, the grain boundaries in sample T3 are oriented with a set grain in a similar orientation. The grain in Sample T3 has lesser dendritic formation compared to that in sample T1. The optical micrographs shown in Figure 10 depict the particle distribution in various zones in different types of samples.

**Figure 11:**
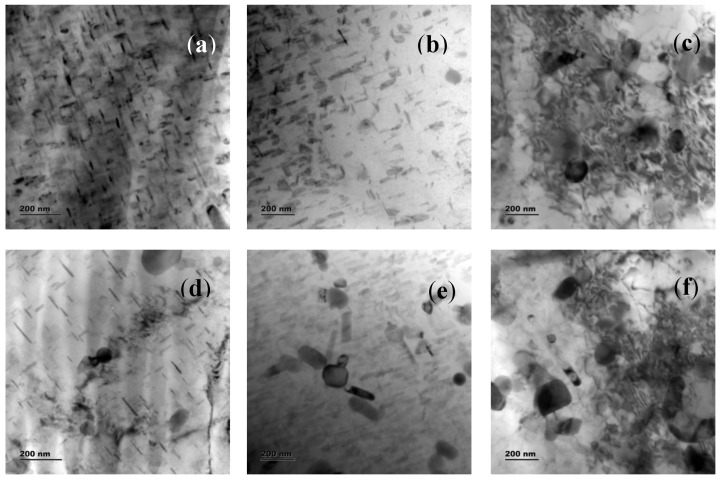
TEM images, (**a**) ultrafine grains in sample T1, (**b**) disc-shaped precipitates in sample T2, (**c**) hybrid particle cluster with precipitates in sample T3, (**d**) Mg_2_Si precipitates in sample T1, (**e**) Hybrid particles in sample T2, and (**f**) Hybrid reinforcement cluster in sample T3.

Figure 11a–f shows the TEM images of different samples. Dislocations are developed when a substance is distorted. The grains in the stir zone go through a recrystallization and deformation cycle before being forged at the tool’s back [47]. The microstructure of the grains formed during the early phases of FSP coarsens, and more dislocations are generated during subsequent thermo-mechanical deformation in the larger grains that preferentially bear load [56]. Equiaxed grains with an average grain size of about 2.2 µm were generated during intense plastic deformation, comparable to the Nugget Zone, providing appropriate deformation heat and facilitating the dynamic recrystallization process [55,58]. Dynamic Recovery (DRV) happens quickly when metals with high stacking fault energy, such as Aluminum, are hot worked. As dislocations grow and interact during the early stages of deformation, the flow stress rises. Dislocations begin to rearrange and produce low-angle boundaries as dislocation density increases and sub-grains form, and the recovery rate accelerates [54]. As dislocation multiplies and recovery occurs due to dislocation rearrangement, the flow stress saturates and approaches a dynamic equilibrium. The flow stress remains constant as the strain grows, resulting in steady-state deformation [58,59,60]. The steady state is mirrored by equiaxed sub-grains with virtually dislocation-free interiors, constant sub-grain size, and sub-grain border misorientation, as shown in Figure 8. These low-angle boundaries evolve into high-angle boundaries during a continual dynamic recrystallization process, resulting in a fine-grained structure [61,62,63]. Regardless of composition, the extreme turbulence of the material in a semi-solid state around the pin causes the particles in the SZ to fragment [33,64].

Grain development kinematics are greatly influenced by the curvature radius and grain borders’ mobility. Due to the pressure differential across the border, grain boundaries tend to move towards the center of curvature [65]. An equilibrium condition can be established when grain boundaries are straight, the angle at the triple points is close to 120, and such a microstructure will withstand more heat cycles. As demonstrated in Field Emission Scanning Electron Microscopy pictures (Figure 10), the grains in the friction stir processed (FSPed) material is equiaxed with clearly defined straight grain boundaries in the current work, and the banded contrast observed shows that the boundaries are in equilibrium [66,67]. Mg_2_Si precipitates are formed and represented in the disc (plate-shaped β precipitate), as shown in Figure 11a–f.

From the microstructure, it can be understood that reinforcement particles are distributed inside the Al-matrix and the likelihood that various defects will be created. The incorporation of hybrid ceramic particles produced a homogeneous zone in SZ, as seen in Figure 10a through Figure 10c. According to microstructural investigations, significant grain structural refinements were seen during FSP, which can be linked to the occurrence of several dynamic recrystallization (DRX) mechanisms.

## 4. Conclusions

- The Al hybrid surface composites were successfully manufactured. The different mechanical characterization tests (tensile, microhardness, and wear) were performed, then detailed microstructure investigations were performed using SEM, OM, and TEM techniques. The most critical outcomes are as follows:
- FSP performs a significant role in grain refinement in the stir zone of processed samples. The EDS analysis confirmed the presence of the reinforcement particles in the composites.
- The increase in hybrid reinforcement content from 5 wt% to 15 wt% enhanced tensile strength and Young’s modulus while decreasing elongation, which implies that the structure became more brittle., i.e., Sample T3 exhibited the highest tensile strength with a reduction in elongation, compared to samples T1 and T2. The ductile fracture occurred in sample T1 due to the deeper dimples observed in fracture SEM images, which was the result of higher Elongation (%) compared to that in samples T2 and T3.
- The stir zone’s microhardness was studied. The heat-affected and the thermomechanical-impacted zones were analyzed, and the stir zone exhibited a higher hardness due to grain refinement. Sample T3 exhibited the lowest wear rate due to the increased hardness compared to samples T1 and T2. The worn surfaces exhibited adhesion wear. Wear grooves were observed with debris.
- SEM and TEM micrographs revealed the hybrid particles’ existence and bonding, which are the main reasons for the increase in hardness.
- The data presented in this study will be useful in developing several hybrid surface composites for future aerospace and automotive applications.

## Figures and Tables

**Table 1:** Elemental composition of Al-Mg_2_-Si alloy.

Element	Aluminum (Al)	Magnesium (Mg)	Silicon (Si)	Iron (Fe)	Zinc (Zn)	Manganese (Mn)	Titanium (Ti)	Copper (Cu)	Chromium (Cr)
Weight Percentage	97.9 to 99.3	0.35 to 0.60	0.30 to 0.60	0.1 to 0.3	0 to 0.15	0 to 0.10	0 to 0.10	0 to 0.10	0 to 0.050

**Table 2:** Detailed composition of the hybrid surface composites.

Sample	Hybrid Reinforcement	Matrix
	B_4_C + SiC + CaCO_3_ (wt%)	Al-Mg_2_-Si (wt%)
T1	5	95
T2	10	90
T3	15	85

## Data Availability

The data that support the findings of this study are available on request from the authors.
